# No clear evidence of neuropathy among patients with high risk for the development of prediabetes/diabetes—a pilot study

**DOI:** 10.3389/fendo.2024.1302013

**Published:** 2024-01-30

**Authors:** Anna E. Körei, Magdolna Békeffy, Adrienn Menyhárt, Karola Osgyán, Ildikó Istenes, Viktor J. Horváth, Péter Kempler

**Affiliations:** Department of Internal Medicine and Oncology, Semmelweis University, Budapest, Hungary

**Keywords:** diabetes, prediabetes, increased risk of diabetes, autonomic neuropathy, sensory neuropathy

## Abstract

**Introduction:**

Autonomic and sensory neuropathy have been observed in both prediabetes and manifest diabetes mellitus. However, there is a lack of available data regarding whether patients at a moderate or high risk of developing diabetes, yet without a current diagnosis of prediabetes or diabetes, exhibit an increased prevalence of neuropathy.

**Methods:**

FINDRISC (Finnish Diabetes Risk Score) was used to classify individuals at risk (≥12 points, *n* = 44; control <12 points, *n* = 28). HbA1c levels >5.6% served as exclusion criteria, and patients with known medical conditions predisposing to neuropathy were also excluded. Cardiac autonomic function (Ewing tests) and peripheral sensory neuropathy (Neurometer and Q-sense) were assessed by standardized protocols, and their potential association with increased FINDRISC points was analyzed using a regression model.

**Results:**

Mean age was 46.7 ± 14.3 years in the control and 55.7 ± 14.1 years in the increased risk group. Male/female ratio did not differ. Individuals with increased risk of diabetes were more obese (BMI: 29.9 ± 12.5 kg/m^2^ vs. 25.9 ± 8.9 kg/m^2^). Additionally, hypertension was more frequent among them (68.2% vs. 17.9%), and their lipid parameters were also less favorable. Parasympathetic neuropathy was present in both groups (56.8% vs. 32.1%, respectively). Sympathetic neuropathy was not found. Sensory nerve dysfunction was of low prevalence in the high-risk group and did not occur in healthy controls. In multiple logistic regression analysis, HbA1c exhibited an independent association with parasympathetic neuropathy (OR: 5.9; 95% CI: 1.08–32.68; *p* < 0.041).

**Discussion:**

An increased risk of developing prediabetes/diabetes does not appear to have a strong correlation with an increased likelihood of developing autonomic or sensory neuropathy. However, the etiology behind the occurrence of parasympathetic autonomic neuropathy in healthy individuals remains unknown.

## 1 Introduction

The global prevalence of prediabetes and diabetes mellitus is rapidly increasing (1, 2). The two conditions are strongly interrelated with up to 70% of individuals diagnosed with prediabetes progressing to diabetes (3). Both diabetes and prediabetes are associated with an increased risk of developing sensory (DSPN) and cardiac autonomic (CAN) neuropathy (3–6), both of which are independent risk factors for mortality in diabetic patients (7, 8).

Although the diagnosis of incident diabetes or prediabetes is based on laboratory tests, identifying individuals at a higher risk of developing diabetes is also possible with non-invasive approaches (3). The FINDRISC (Finnish Diabetes Risk Score) questionnaire stands out as widely utilized. It demonstrates the capacity to predict the onset of incident diabetes with an acceptable sensitivity and specificity (9). Non-invasive models for predicting diabetes include mostly similar risk factors, but they weigh the components differently. Notably, an elevated fasting plasma glucose level within the normoglycemic range emerges as an independent risk factor for type 2 diabetes in young men (10). In addition, increased glucose values were observed as early as 13 years prior to the diagnosis of diabetes (11). These data indicate that patients with normal blood glucose levels but at higher risk for diabetes may exhibit an altered metabolic status, potentially representing an increased susceptibility to microvascular complications as well. Also, certain risk factors in non-invasive prediction models [e.g., age, higher body mass index (BMI), high blood pressure] are associated with the development of neuropathy even in the absence of abnormal glucose metabolism (12). As high-risk diabetes, prediabetes, and manifest diabetes represent a glycaemic continuum and the latter two entities are clearly associated with different types of neuropathies, our study aimed to determine whether an increased risk of diabetes is associated with a higher prevalence of neuropathy or neuropathy-related symptoms.

## 2 Materials and methods

### 2.1 Setting and participants

We enrolled adult patients (aged 18 years and older) who attended the diabetes and general medicine outpatient clinic of the Department of Internal Medicine and Oncology, Semmelweis University from 2020 to 2023. The clinic serves as a secondary referral center for a suburban area of Budapest, Hungary, with approximately a hundred thousand inhabitants. We included patients referred to our clinic by general practitioners, exhibiting an increased risk of developing type 2 diabetes (FINDRISC ≥12 points) but no known history of glucose metabolism abnormalities.

This group of patients served as our primary focus. Patients referred with other, general health problems unrelated to any glucose metabolism abnormalities were included in the control group (FINDRISC <12 points). HbA1c levels higher than 5.6% (and fasting blood glucose equal or higher than 5.9 mmol/l) served as exclusion criteria and patients with diseases known to cause neuropathy were also excluded from further investigation. Furthermore, to exclude any potential glucose metabolism abnormalities, a 75-g oral glucose tolerance test was administered to patients with a FINDRISC ≥12 points. In addition to an assessment of CAN and DSPN, demographic data, anthropometrics, lifestyle information (including smoking habits and alcohol consumption, eating habits, and physical activity), current and previous chronic illnesses, current (temporary or continuous) medications and laboratory data were collected on a standardized data entry form. The study protocol was approved by the local ethical committee, and all participants gave written informed consent.

### 2.2 Definition of DSPN and CAN

Subclinical and clinical DSPN and CAN were diagnosed in accordance with the Toronto Diabetic Neuropathy Expert Group recommendation (13) and were performed using standardized protocols (7, 14, 15). DSPN was evaluated by Neurometer CPT (Neurotron Inc., Baltimore, MD, USA), Medoc Q-Sense device (Ramat Yishay, Israel), calibrated 128-Hz tuning fork, and Semmes-Weinstein monofilament. Current perception threshold (CPT) was measured at the median and peroneal nerves (digital branches) by the Neurometer device at three different frequencies (2000 Hz, 250 Hz, and 5 Hz) assessing large myelinated, small myelinated and small unmyelinated sensory fiber function, respectively. Cold and heat detection thresholds were assessed using a thermal sensory analyser from the Q-Sense instrument. Diagnosis of DSPN was confirmed when abnormal results were found bilaterally using the same diagnostic method.

Symptoms consistent with painful diabetic neuropathy were assessed using the Neuropathy Total Symptoms Score–6 (NTSS-6) questionnaire.

CAN was assessed by the standard cardiovascular reflex tests (CARTs) using Ewing’s battery (13), except the handgrip test (15). Briefly, the parasympathetic function was assessed by heart rate responses to deep breathing and standing (30:15 ratio) and by performing the Valsalva maneuver (Valsalva ratio). The results of the deep-breathing test were expressed as the difference of the highest heart rate during inspiration and the lowest heart rate during expiration (beat-to-beat variation; beats/min). Sympathetic autonomic function was assessed by blood pressure responses to standing (orthostatic hypotension test). Diagnosis of CAN was defined as the presence of at least two pathological reflex test results, or alternatively, one that is clearly abnormal and two that fall within the borderline range. Patients taking antihypertensive medications (especially beta-blockers and non-dihidropyridin type Ca-antagonist), which could affect CART results derived from analysis of heart rate changes, were requested to stop medications at least 24h prior to investigations. Patients were also asked to avoid consuming coffee, other caffeinated beverages, alcohol, smoking, and strenuous exercise at least 6h before testing.

Consistent with recent guidelines, age-related reference values were used for the interpretation of heart rate–based tests. To evaluate severity of CAN, an autonomic neuropathy score (CAN score) was obtained by scoring the results of the reflex tests: 0, 1, and 2 for normal, borderline, and abnormal test results.

### 2.3 Measuring other variables

After we determined eligibility including sex at birth and age, height and weight were measured by calibrated instruments. Smoking burden was defined as package year. Weekly alcohol consumption (beer, wine, and spirits) was recorded and considered to be high if the level of consumption was >14 units/week. All the laboratory measurements were completed by using standardized protocols of the Department of Laboratory Medicine, Semmelweis University.

Available medical records were screened for the following medical conditions: a possible previous documentation of diabetes, diagnosis of hypertension and hyperlipidaemia, previous major cardiovascular event (including myocardial infarction, heart failure, peripheral vascular disease, and cerebrovascular accident), known dementia, thyroid disease, chronic obstructive pulmonary disease, connective tissue diseases, inflammatory bowel disease, chronic liver disease, chronic kidney disease (CKD III–V), chronic anaemia, radiologically confirmed vertebral disc herniation, and malignancies. Information collected from medical records was supplemented with direct information from the participants.

### 2.4 Statistical analysis

The Kolmogorov–Smirnov test for normality was performed on all variables. Normally distributed data were presented as means ± SD, while non-normally distributed data, such as triglycerides, cholesterol, blood pressure, and sensory and autonomic measures were expressed as median (interquartile range). Categorical data were reported as *n* (%).

For comparison between groups, Mann-Whitney U-test or paired t-test for continuous variables and χ^2^ test for categorical data were used based on the variable’s normality of distribution. To investigate the explanatory variables of cardiovascular autonomic neuropathy (CAN) (only parasympathetic autonomic dysfunction was detected within the study population), a multiple logistic regression model was employed using cardiovascular parasympathetic autonomic neuropathy as dependent variable. All variables that exhibited a univariate association (*p* < 0.1) with parasympathetic autonomic neuropathy were included in the analysis. Consequently, the model incorporated age, the presence of hypertension as a comorbidity, HbA1c, and the risk of developing type 2 diabetes represented by the total score of FINDRISC questionnaire. Explanatory factors of sensory impairment could not be analyzed due to the very limited occurrence of sensory neuropathy as outcome measure within this study population. *P* < 0.05 was considered as statistically significant. All analyses were performed using SPSS version 27.0.

## 3 Results

### 3.1 Baseline parameters

A total of 44 patients with an increased risk of diabetes (FINDRISC ≥12 points, referred as high-risk group) were investigated, along with 28 control patients (FINDRISC <12 points, referred as control group). The total score difference between the two groups was approximately 10 points, indicating a significant disparity. Participants of the high-risk group were older; their weight, waist, and hip circumference was higher; their height was lower; and their BMI was also significantly higher. On the other hand, the waist-to-hip ratio (WHR) did not differ significantly and no gender difference could be seen. Hypertension was more prevalent in the high-risk group, leading to a higher frequency of antihypertensive medication usage. There was no difference in the use of non-steroidal anti-inflammatory drug medication between the two groups. Family history of diabetes and hypertension was also more frequent among members of the high-risk group. There was no difference in the smoking habit and alcohol consumption (>14 units/week) between the two groups.

### 3.2 Measured parameters

Average diastolic blood pressure was higher in the high-risk group, but no difference was found in systolic blood pressure and resting heart rate. HbA1c was mild; fasting blood glucose was considerably higher (both significantly different) in the high-risk group. Low-desity lipoprotein (LDL; measured) and high-density lipoprotein (HDL) did not exhibit differences; however, there were significant disparities in total cholesterol, triglyceride levels, and triglyceride/HDL ratio, all of which were higher in the high-risk group. No difference in kidney function and sedimentation rate was detected between the two groups.

### 3.3 Neuropathy parameters

#### 3.3.1 Parameters of CAN

Beat-to-beat variation being a sensitive marker for parasympathetic neuropathy was significantly lower, and cardiovascular autonomic score was significantly higher in the high-risk group when compared to healthy controls. Parasympathetic neuropathy occurred more frequently among patients with a moderate to high risk of developing diabetes. The 30:15 ratio and the Valsalva ratio (the latter controlled partially by sympathetic nerves) remained within the normal range in both groups, and no cases of sympathetic neuropathy were diagnosed in either the high-risk or control group.

#### 3.3.2 Parameters of DSPN

Sensory neuropathy only occured in the high-risk group, but in only eight patients. Regarding the symptoms associated with DSPN, sensations of pricking and lancinating nature were more frequently reported in the high-risk group. However, there was no difference in the frequency of aching and burning pain, numbness, and allodynia between groups. Regarding NTSS, there was a trend toward higher scores in the high-risk group, but the difference did not reach statistical significance. Similarly, protective sensation assessed by the monofilament in the lower limbs was found not significantly different between the high-risk and healthy control groups. In contrast, reduced vibration sensation as measured with a tuning fork was found in the high-risk group compared to healthy control subjects with the differences being significant on all extremities except the right arm. Comparing CPTs between groups, a significant difference was detected only in CPTs of large and small myelinated nerve fibers of the median nerves. However, assessing CPT abnormality between high risk for diabetes and healthy control groups, a pathological CPT was detected more frequently in the large myelinated fibers of the peroneal nerve in the high-risk group.

Finally, when comparing thermal sensation thresholds between groups, warm sensation thresholds were found significantly higher in the high-risk group. Regarding cold thermal sensation, high-risk patients had consistently lower cold detection thresholds on all limbs. Yet, the differences were found significant only for thresholds measured on the lower limbs.

The main features of the study participants are shown in **Tables 1**–**4**.

**Table 1:** Anthropometric and clinical characteristics of the participants.

Patient characteristics	FINDRISC < 12 (*n* = 28)		FINDRISC > 11 (*n* = 44)		*p*
Measured parameter	Average/*n*	SD/IQR/%	Average/*n*	SD/IQR/%	
Age (years)*	46.7	± 14.3	55.7	± 14.1	**0.011**
Weight (kg)*	78.9	± 14.4	89.3	± 20.6	**0.024**
Height (cm)*	175	± 0.1	169	± 2.1	**0.009**
BMI (kg/m^2^)*	25.9	± 8.9	29.9	± 12.5	**< 0.001**
Waist circumference (cm)	95	79–101	102	91.3–110.8	**0.014**
Hip circumference (cm)	100	93.5–110.3	111	103.5–116.3	**0.004**
WHR	0.95	0.87–0.99	0.94	0.83–0.98	0.904
Smoking (pack year)	0	0–0	0	0–0	0.171
Systolic blood pressure (mmHg)	129	118.5–136.8	128	122–110	0.432
Diastolic blood pressure (mmHg)	79	75–82	86	76–92	**0.046**
Resting heart rate (bpm)	74	67.3–80	68	62–75	0.6
Fasting glucose (mmol/l)	4.8	4.6–5.2	5.5	5.2–5.9	**< 0.001**
HbA1c (%)*	5.2	± 0.25	5.4	± 0.2	**0.029**
Triglyceride (mmol/l)	1.3	0.8–1.79	1.51	1.1–2.05	**0.033**
Cholesterol (mmol/l)	4.76	4.13–5.1	5.5	4.5–6	**0.004**
LDL cholesterol (mmol/l)	3.1	2.4–3.5	3.3	2.8–4.2	0.058
HDL cholesterol (mmol/l)	1.5	1.2–1.7	1.3	1.0–1.6	0.226
TG/HDL ratio	2.2	1.04–2.99	2.61	1.7–4.7	**0.039**
Uric acid (µmol/l)	303	286–338	329	280–403	0.116
Blood urea nitrogen (mmol/l)	5.2	4.2–6.7	5.5	4.6–6.7	0.591
Creatinine (µmol/l)	80	69–90	76	68–87	0.583
We (mm/h)	7.5	6–11	7.0	5.5–17.5	0.803
Red blood cell count (G/l)	4.79	4.36–5.1	4.75	4.6–5.3	0.933
TSH (mIU/l)	1.395	1.08–1.75	1.43	0.82–1.97	0.98
FQ total score	6	3.3–8	16.5	13–19	**< 0.001**
NTSS	0	0–1	1	0–2	**0.017**
Male	16	57%	23	52%	0.686
Hypertension	5	17.9%	30	68.2%	**< 0.001**
Beta blocker	2	7.1%	18	41.9%	**0.001**
RAAS blocker	3	10.7%	24	55.8%	**< 0.001**
Diuretic	0	0.0%	12	27.9%	**0.002**
Calcium channel blocker	2	7.1%	9	20.9%	0.181
NSAID	2	7.1%	5	11.5%	0.696
Alcohol usage (>14 U/week)	0	0.0%	0	0.0%	1
Family diabetes negative	23	82%	15	34%	
Family diabetes second degree	4	14.30%	12	27.30%	
Family diabetes first degree	1	3.60%	17	38.60%	
Family diabetes/negative					**< 0.001**
Family hypertension negative	16	61.50%	11	26.20%	
Family hypertension second degree	10	38.50%	17	40.50%	
Family hypertension first degree	0	0	14	33.30%	
Family hypertension/negative					**< 0.001**

Between group comparisons were carried out by Mann-Whitney U-test. Normally distributed data (*) were compared with paired t-test. Normally distributed data: mean ± SD; not normally distributed: median and interquartile range (IQR). Categorical data compared with and χ2 test and expressed as n (%). BMI, body mass index; WHR, waist-to-hip ratio; FQ total score, FINDRISC questionnaire total score; NTTS, neuropathy total symptom score. RAAS, renin-angiotensin-aldosteron; NSAID, non-steroidal anti-inflammatory drug.Bold refers to significant differences.

**Table 2:** Cardiac autonomic (CAN) markers of the different populations (unadjusted data).

CAN parameters	FINDRISC <12 (*n* = 28)		FINDRISC > 11 (*n* = 44)		*P*
Measured parameter	Average/*n*	SD/%	Average/*n*	SD/%	
Beat to beat variation	17	8.1	11.5	15.2	**0.008**
Beat to beat score	0	0.0	1	3.4	**0.002**
Valsalva ratio	1.8	1.1	1.9	2.0	0.641
Valsalva score	0	0.0	0	0.0	0.181
30:15 ratio	1.07	0.2	1.05	0.2	0.666
30:15 score	0	0.0	0	0.0	0.387
Orthostatic hypotension (mmHg)	6	16.2	2	6.8	0.685
Autonomic score	1	2.7	3	6.8	**0.005**
Parasympathetic neuropathy	9	32.1%	25	56.8%	**0.035**
Sympathetic neuropathy	0	0.0%	0	0.0%	1

Parasympathetic and sympathetic function is considered to be pathological based on the Toronto Diabetic Neuropathy Expert Group recommendation (13). [Mean ± SD for continuous variable; n (%) for categorical parameters.].Bold refers to significant differences.

**Table 3:** Results of peripheral sensory neural function measurement and neural sensation alterations in the different groups (unadjusted data; mean ± SD for continuous.

DSPN parameters (as measured)	FINDRISC < 11 (*n* = 28)		FINDRISC > 11 (*n* = 44)		
Measured parameter	Average	SD	Average	SD	*p*
Monofilament left leg	6	16.2	5	16.9	**0.045**
Monofilament right leg	5	13.5	5	16.9	0.146
Tuning fork left arm	8	21.6	7	0.1	**0.004**
Tuning fork right arm	8	21.6	7	0.1	0.113
Tuning fork left leg	8	21.6	7	23.7	**0.001**
Tuning fork right leg	8	21.6	7	23.7	**< 0.001**
Medianus 2000 Hz left	244	658.7	305	1032.2	**0.006**
Medianus 2000 Hz right	244	658.7	329.5	1115.1	**0.012**
Medianus 250 Hz left	76	205.2	114.5	387.5	**0.029**
Medianus 250 Hz right	83	224.1	118.5	401.0	**0.029**
Medianus 5 Hz left	54	145.8	66	223.4	0.188
Medianus 5Hz right	52	140.4	67	226.7	0.355
Peroneal 2000 Hz left	347	936.8	345	1167.6	0.576
Peroneal 2000 Hz right	373	1007.0	339.5	1149.0	0.648
Peroneal 250 Hz left	135	364.5	156.5	529.6	0.269
Peroneal 250 Hz right	131	353.7	142	480.6	0.283
Peroneal 5 Hz left	131	353.7	94	318.1	0.818
Peroneal 5 Hz right	100	270.0	109	368.9	0.393
Warm left arm	33.7	91.0	34.5	116.8	**0.002**
Cold left arm	30.7	82.9	30.5	103.2	0.174
Warm right arm	33.7	91.0	34.7	117.4	**0.025**
Cold right arm	30.7	82.9	30.6	103.6	0.373
Warm left leg	37.6	101.5	39.5	133.7	**0.01**
Cold left leg	30	81.0	28.6	96.8	**0.005**
Warm right leg	37.9	102.3	33.6	12.9	**0.014**
Cold right leg	29.6	79.9	28.9	97.8	**0.03**
NTSS aching	1	3.60%	7	16.30%	0.259
NTSS burning	1	3.60%	4	9.30%	0.642
NTSS prickling	3	10.70%	12	27.90%	**0.031**
NTSS numbness	7%	25%	15	34.90%	0.154
NTSS lancinating	0	0	5	11.60%	**0.018**
NTSS allodynia	0	0	3	7%	0.074

NTTS, neuropathy total symptom score.

*n* (%) for categorical parameters).Bold refers to significant differences.

**Table 4:** Parameters determine the presence or absence of parasympathetic cardiac autonomic neuropathy in both groups.

Measured parameter	PSY CAN present	PSY CAN absent	*P*
HbA1c (%)	5.5 ± 0.2	5.2 ± 0.2	**0.05**
Age (years)	56.7 ± 14.6	48.2 ± 13.9	**0.03**
BMI (kg/m^2^)	29.5 ± 6.5	28.3 ± 4.8	0.23
WHR	0.92 ± 0.1	0.90 ± 0.1	0.83
tg (mmol/l)	1.8 ± 1.1	1.5 ± 0.6	0.85
Total chol. (mmol/l)	5.3 ± 1.2	5.0 ± 1.1	0.41
HDL (mmol/l)	1.4 ± 0.4	1.3 ± 0.4	0.41
kreat (µmol/l)	82 ± 36	76 ± 13	0.20
FQ total score (point)	13.6 ± 5.9	10.6 ± 5.7	**0.03**
Hypertension	21 (62%)	14 (34%)	**0.05**
Male	15 (44%)	24 (63%)	0.16

Parameters found to be significantly different were further incorporated to regression model. PSY CAN, parasympathetic cardiac autonomic neuropathy; BMI, body mass index; WHR, waist-to-hip ratio; FQ total score, FINDRISC questionnaire total score.Bold refers to significant differences.

### 3.4 Association between diabetes risk and the presence of cardiovascular autonomic and sensory neuropathy

In order to explore the explanatory variables for cardiovascular autonomic neuropathy CAN, clinical and laboratory data of study participants with and without CAN were compared (**Table 4**). Significant differences between groups were detected in respect of age, HbA1c, the presence of hypertension as comorbidity, and the total score obtained in the FINDRISC questionnaire reflecting type 2 diabetes risk. No significant differences between participants with and without CAN were found regarding BMI; WHR; serum lipid levels, such as triglyceride (TG); total cholesterol; HDL; and serum creatinine. In multiple logistic regression analysis, glycaemia (indicated by HbA1c levels) emerged as the only and independent factor associated with CAN (OR: 5.9; 95% CI: 1.08–32.68; *p* < 0.041) (**Table 5**).

**Table 5:** Association between the presence of parasympathetic CAN and their determinants.

	ExpB	Upper 95% CI	Lower 95% CI	Sig
HbA1c	5.938	1.079	32.678	0.04
Age	1.017	0.974	1.062	0.44
HT	1.165	0.323	4.195	0.82
FQ total score	1.035	0.933	1.148	0.52

After adjusting the existence of parasympathetic CAN in the control and high-risk group to HbA1c, age, hypertension, and FQ total score. The difference of the frequency of parasympathetic CAN between the two groups remained no more significant. Majority of the difference could be explained by the difference of HbA1c between the two groups. FQ total score: FINDRISC questionnaire total score.

Variables underlying sensorimotor neuropathy could not be assessed given their low incidence rate observed in this study.

## Discussion

The main objective of this study was to evaluate whether patients with an increased risk for type 2 diabetes (FINDRISC ≥ 12 points) without clinically manifest glucose metabolism disorders have an increased prevalence of neuropathy. CAN is more frequent in patients with an increased risk of developing type 2 diabetes when compared to healthy control subjects, and it shows independent association with HbA1c levels within the normal range. As for sensorimotor polyneuropathy, only some slight alterations hinting to early DSPN could be verified in high-risk patients. However, parasympathetic neuropathy was documented in both the control and the increased risk groups. This finding needs further attention.

It is reasonable to assume that patients at risk of developing diabetes or prediabetes (without clinically manifested glucose metabolism disorder) may exhibit various forms of neuropathy with variability in their characteristics (4). Higher mean fasting plasma glucose levels within the normoglycemic range in young individuals is an independent risk factor for type 2 diabetes (10). Additionally, increased glucose values were observed in the Whitehall II study as early as 13 years before the diagnosis of diabetes (11). Although we have no direct evidence that the FINDRISC questionnaire identifies these people, we cannot exclude it. Some pathophysiological aspects may support our findings on slight autonomic and sensory dysfunction in patients being at higher risk for type 2 diabetes. Enhanced polyol pathway activity and increased oxidative and nitrosative stress are well-known mechanisms related to the development of neuropathy and are associated with hyperglycaemia (16). Higher quartile of glucose (or HbA1c) levels even within the normal range might have an impact on neuronal function in high-risk individuals.

In the present study, glycaemia within the normal range emerged as the only independent factor associated with CAN. Age and hypertension are addressed by the FINDRISC questionnaire itself and, assumably, their effects were eradicated by incorporating the score results into the regression model. Glycaemia, age, and hypertension are proven risk factors of autonomic neuropathy (4, 17). Similarly to the present study, glycaemia was found the only independent explaining measure for sensory nerve dysfunction, as observed in our previous study involving patients with impaired glucose tolerance (14).

We assessed CAN by standard CARTs. CARTs are still the gold standard of CAN assessment. Short-term heart rate variability (HRV) indices might be additional resources being easier, perhaps more sensitive and patient independent compared to CARTs (18). Also, primary sympathovagal imbalance was suggested a common pathway to the development of hypertension and diabetes mellitus (19). Large epidemiological studies also support the hypothesis that autonomic dysfunction may predict the development of future type 2 diabetes (5). Orthostatic hypotension is a late and insensitive marker of CAN. Accordingly, no instances of sympathetic CAN could be detected in this study. One can speculate that sudomotor tests would provide a more precise assessment of CAN in our study. However, no strong evidence on diagnostic accuracy of sudomotor function assessment in prediabetic and diabetic sympathetic neuropathy is available (18).

It should also be noted that a limited number of patients (*n* = 9; 32.1%) had parasympathetic neuropathy in the control group. It may be associated with the high prevalence of cardiovascular risk factors (hypertension, hyperlipidaemia, etc.) being also known as predictors of CAN. The presence of an early stage neuropathy in, otherwise, healthy individuals underscores the importance of investigating factors that may contribute to the development of neuropathy.

The assessment of peripheral neural dysfunction with the Neurometer is also a matter of debate. We have three decades of experience with this instrument (14, 20–30), and it seems to be a reliable and reproducible method to detect early changes of sensory dysfunction. Our recent meta-analysis also confirmed its usefulness and comparability with other sensory assessment tools (8). Using the Neurometer and quantitative sensor testing (Q-Sense), only very slight differences between individuals at high risk of type 2 diabetes and healthy controls could be observed: high-risk patients tended to have diminished vibration, current, and thermal perception with the thresholds remaining in the normal reference ranges. Whether these changes have clinical significance or predict future decline in sensory function remain open. Gold standard methods of small-fiber neuropathy such as CCM (corneal confocal microscopy) and punch biopsy may be more sensitive (31). However, CCM and IENFD (intraepithelial nerve fiber density) assessments through punch biopsy were not accessible to us during the study. Furthermore, the clinical utility of IENFD assessment is limited by its invasive nature.

In this study, the risk of diabetes was assessed using the FINDRISC questionnaire (32–35). The FINDRISC is able to identify not only diabetes but also milder forms of glucose abnormalities depending on the cutoff point chosen (32, 33, 35). In our study, we opted for a cutoff value of >11 points to identify patients at a moderate to high risk of developing diabetes, a threshold slightly lower than the cutoff values used by most studies (>13 and >15 points). However, in a previous study, lowering the cutoff value to ≥11 in the FINDRISC questionnaire yielded an enhanced sensitivity of 73%, a specificity of 67%, and an excellent negative predictive value of 98.5% for detecting diabetes (34). Moreover, a cross-sectional analysis of the National Health and Nutrition Examination Survey spanning 1999–2010 recommended an optimal cutoff point of 10 for men and 12 for women in detecting diabetes and 9 for men and 10 for women in detecting prediabetes (35). Considering our aim to assess the neuropathy burden of individuals at a higher risk of diabetes yet without prediabetes or diabetes, the chosen cutoff value of >11 seemed to be appropriate. The score of >11 was also shown to be a reasonable cutoff for the optimal utilization of OGTT in screening for abnormal glucose metabolism in the population (35).

Certain limitations of the present study need to be addressed. An important limitation is the small sample size that restricts our ability to adjust our data for potentially confounding factors and conduct subgroup analyses. A further important confounding factor is the usage of beta-blockers. Nonetheless, patients followed our instructions to discontinue heart rate–modifying drugs 24h before the investigation. Usage of diuretics would primarily affect the orthostatic hypotension test. In our study, no participant had orthostatic hypotension and, therefore, the clinically significant effect of diuretics can be excluded. Due to our inability to continuously monitor glucose concentration (even for a limited duration), no precise assessment of the effect of total glycaemic burden on neural function was possible and further investigations are required to explore this aspect in detail. Given the cross-sectional design of the present study, no causative relationship between CAN and HbA1c could be concluded. The overall atherosclerotic cardiovascular risk was low or moderate among the patients and, therefore, we did not collect information on the integrity of the coronary circulation, although the presence of coronary disease could potentially influence our results. As our cohort included participants referred to a university center, referral bias cannot be excluded. We assessed obesity by BMI and waist circumference, but these parameters do not address the visceral fat tissue, which is associated with an increased risk of metabolic syndrome, diabetes, and cardiovascular diseases (36) potentially leading to neuropathy.

Our study has notable strengths as well. This was the first attempt to demonstrate an association between an increased risk of type 2 diabetes risk and the presence of neuropathy. The utilization of gold standard measures of diabetic autonomic neuropathy and validated instruments for sensory neuropathy assessment constitutes a valuable strength rendering our study easily reproducible. The fact that all tests and examinations were conducted in the same laboratory, using the same methodology for both groups reduces the risk related to the methods applied. Key risk factors for neuropathy were systematically collected at the time of patient selection. Additionally, electronic medical records were available for all patients, ensuring comprehensive data retrieval without missing values.

In summary, our study did not demonstrate clear evidence of an elevated risk of autonomic and sensory neuropathy among patients with an increased risk of developing diabetes but without a manifest glucose metabolism disorder (FINDRISC >11 points). However, the more common presence of parasympathetic neuropathy and subtle sensory alterations found in this patient population underscores the need for further investigations to clarify the factors contributing to the development of parasympathetic and sensory dysfunction and warrants further prospective studies to reveal their clinical significance and long-term consequences.

## Data availability statement

The original contributions presented in the study are included in the article/supplementary material. Further inquiries can be directed to the corresponding author.

## Ethics statement

The studies involving humans were approved by Semmelweis University Regional and Institutional Committee of Science and Research Ethics. The studies were conducted in accordance with the local legislation and institutional requirements. The participants provided their written informed consent to participate in this study.

## Author contributions

AK: Data curation, Formal analysis, Investigation, Writing – review & editing. MB: Data curation, Investigation, Writing – review & editing. AM: Data curation, Investigation, Writing – review & editing. KO: Data curation, Investigation, Project administration, Writing – review & editing. II: Investigation, Writing – review & editing. VH: Data curation, Investigation, Writing – original draft. PK: Conceptualization, Funding acquisition, Methodology, Resources, Supervision, Writing – review & editing.
